# A Synergistic Composite Hydrogel Integrating *Periplaneta americana* Extract and Calamine for Refractory Diabetic Wound Healing

**DOI:** 10.3390/pharmaceutics18050617

**Published:** 2026-05-18

**Authors:** Chenxiao Chu, Xingting Fan, Xiaoman Zhang, Tongyao Zhao, Yuying Wang, Xing Tang, Yu Zhang, Tian Yin

**Affiliations:** 1 Department of Pharmaceutics, Shenyang Pharmaceutical University, Shenyang 110016, China; 2 School of Functional Food and Wine, Shenyang Pharmaceutical University, Shenyang 110016, China; 3 School of Traditional Chinese Materia Medica, Shenyang Pharmaceutical University, Shenyang 110016, China

**Keywords:** *Periplaneta americana*, calamine, composite gel, diabetic wound healing

## Abstract

**Background:** Diabetic foot ulcers (DFUs) are difficult to heal because hyperglycemia-associated pathological exudation, excessive oxidative stress, chronic inflammation, and impaired cellular regeneration jointly maintain a nonhealing wound microenvironment. This study aimed to develop and evaluate a composite hydrogel containing *Periplaneta americana* (PA) extract and calamine as a Zn^2+^ source for dynamic modulation of the diabetic wound microenvironment and promotion of tissue repair. **Methods:** A PA composite hydrogel was prepared and assessed in vitro for reactive oxygen species (ROS)-scavenging activity and effects on fibroblast migration. Therapeutic efficacy was further evaluated in a streptozotocin (STZ)-induced diabetic full-thickness wound model in rats. Wound closure, histological remodeling, oxidative stress markers, inflammatory mediators, growth factors, angiogenesis, and AGEs-RAGE/NF-κB pathway-related changes were analyzed. **Results:** The composite hydrogel reduced excessive intracellular ROS and enhanced fibroblast migration in vitro compared with pathological-condition controls. In diabetic rats, topical treatment accelerated macroscopic wound closure and promoted more mature histological repair. Mechanistic analyses showed attenuation of the AGEs-RAGE/NF-κB signaling axis, accompanied by restoration of superoxide dismutase activity, reduction of malondialdehyde levels, and suppression of TNF-α-associated inflammatory responses. The improved wound microenvironment was associated with increased expression of platelet-derived growth factor and basic fibroblast growth factor, enhanced cellular proliferation, and increased neovascularization within the wound tissue. **Conclusions:** The PA composite hydrogel improved diabetic wound healing by concurrently alleviating oxidative and inflammatory barriers and enhancing regenerative signaling. These findings suggest that microenvironment-modulating PA composite hydrogel systems may represent a promising therapeutic strategy for refractory diabetic wounds.

## 1. Introduction

Diabetes mellitus (DM) has emerged as a major global health challenge in the 21st century, with its complications placing an immense burden on healthcare systems worldwide [1,2]. Diabetic foot ulcers (DFUs), one of the most severe and common complications, affect up to 25% of diabetic patients during their lifetime [3]. Characterized by impaired angiogenesis, persistent inflammation, and heightened amputation risks, DFUs are notoriously difficult to heal [4]. While the current standard of care—comprising wound debridement, pressure offloading, and standard passive dressings—provides foundational physical management, it fundamentally fails to actively correct the underlying pathophysiological dysfunctions of the DFU microenvironment. Specifically, the pathological accumulation of advanced glycation end products (AGEs) and their engagement with RAGE acting as a persistent upstream trigger, sustaining a toxic microenvironment that standard dressings cannot reverse. Therefore, developing a bioactive topical formulation capable of actively dismantling this pathological cascade is of urgent clinical necessity [5].

*Periplaneta americana* (PA), an ancient insect species with a 350-million-year evolutionary history, has been extensively documented in traditional Chinese medicine for its potent tissue-regenerative, anti-inflammatory, and antimicrobial properties. Modern pharmacological studies have corroborated these effects, demonstrating that PA extracts can robustly promote granulation tissue formation, enhance angiogenesis, and modulate local immune responses [6,7,8,9]. However, the clinical translation of PA in its conventional liquid form (e.g., Kangfuxin Liquid) faces significant challenges in DFU management. Diabetic ulcers are typically characterized by an exudate-rich, hyperglycemic microenvironment that inherently inhibits fibroblast migration and delays epithelial barrier reconstruction [10,11]. Applying a liquid PA formulation to such wounds exacerbates local moisture overload, potentially leading to tissue maceration, stalled scabbing, and poor patient compliance due to volatile aldehyde-induced odors (e.g., hexanal and isovaleraldehyde) [12].

To overcome the inherent mismatch between liquid PA and the highly exudative nature of DFUs, we designed a synergistic formulation strategy by integrating calamine into a biomimetic hydrogel matrix [13,14,15]. Calamine, primarily composed of zinc oxide, acts not only as a potent astringent to absorb pathological exudate and physically isolate external irritants but also serves as a crucial source of bioactive zinc ions (Zn^2+^) [16]. Furthermore, hydrogels present an ideal biomimetic delivery system due to their porous, extracellular matrix (ECM)-like structure [17]. Unlike liquid formulations, hydrogels offer dynamic physical pathogen isolation [18], alleviate neuropathic pain [19], and prolong the retention of active therapeutics [20]. By engineering a hydrogel with optimized shear-thinning properties and enhanced mucoadhesion, we can ensure the sustained release of PA, transforming it from a transient liquid wash into a dynamic, long-acting microenvironment modulator [21,22].

**Figure 1:**
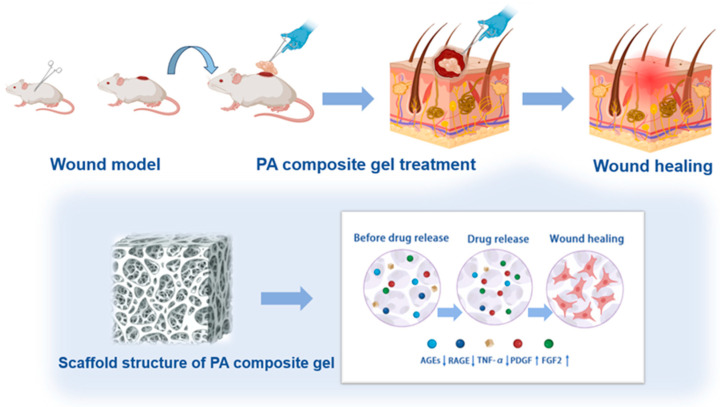
Schematic diagram of PA composite gel for the treatment of diabetic refractory wounds.

In this study, guided by the principle of “synergistic multi-component therapy,” we developed a novel PA-calamine composite hydrogel designed to simultaneously address the physical and biochemical impediments of DFU healing (Figure 1). We hypothesize that this synergistic platform will fundamentally reprogram the hostile wound microenvironment. Specifically, we propose that the interplay between PA bioactives and calamine-derived Zn^2+^ will decisively dismantle the AGEs-RAGE/NF-κB inflammatory axis and neutralize local oxidative stress. Concurrently, this resolution of the toxic microenvironment is expected to amplify the secretion of platelet-derived growth factor (PDGF) and fibroblast growth factor (FGF2). By providing exudate-directed physical protection and multi-target molecular modulation, this composite hydrogel aims to offer a comprehensive, disease-modifying therapeutic paradigm for the clinical management of refractory diabetic wounds.

## 2. Materials and Methods

### 2.1. Materials

*Periplaneta americana* was supplied by Jiangsu Union Soil Breeding Base Co., Ltd. (Nanjing, China). Calamine was purchased from Shangqiu Liangfeng Sanitary Products Co., Ltd. (Shangqiu, China). Carbomer 940 was purchased from MedChemExpress LLC (Monmouth Junction, NJ, USA). Hydroxypropyl-β-cyclodextrin (HP-β-CD) was obtained from Xi’an Deli Biochemical Co., Ltd. (Xi’an, China). Glycerol and sodium carboxymethyl cellulose (CMC-Na) were purchased from Tianjin Hengxing Chemical Reagent Manufacturing Co., Ltd. (Tianjin, China). Gelatin and sodium alginate were purchased from Tianjin Bodi Chemical Co., Ltd. (Tianjin, China). All other reagents were of analytical grade and used as received unless otherwise stated.

### 2.2. Preparation of PA Extract

This study employed a single-factor experimental design to optimize the extraction process of *Periplaneta americana*, with the contents of three marker components (uracil, hypoxanthine, and inosine) as comprehensive evaluation indices. A systematic comparison was conducted between ultrasound-assisted extraction and heat reflux extraction methods. For the ultrasonic-assisted extraction method, critical parameters including extraction time, solid-to-liquid ratio, and ethanol concentration were investigated for their effects on the target component contents. For the heat reflux method, process parameters involving extraction time, temperature, and ethanol concentration were systematically studied.

Taking the representative heat reflux process as an example: Precisely weighed raw material was refluxed with 60% ethanol at 70 °C for 1 h, with periodic solvent replenishment to compensate for evaporation loss. The extract was stirred for a period of time, filtered, and then concentrated under reduced pressure to obtain a dense extract with a relative density of 1.15–1.35.

Quantitative analysis of the marker components was performed using high-performance liquid chromatography (HPLC) under the following conditions: Thermo Hypersil GOLD C18 reversed-phase column (4.6 mm × 250 mm, 5 μm; Thermo Fisher Scientific, Waltham, MA, USA); column temperature 25 °C; detection wavelength 254 nm; flow rate 0.6 mL/min; injection volume 10 μL. The mobile phase consisted of (A) an aqueous solution of acetic acid (pH adjusted to 3.4 ± 0.1 with glacial acetic acid) and (B) methanol. This weakly acidic condition was specifically chosen to suppress the ionization of the marker components (hypoxanthine and inosine), thereby preventing peak tailing and optimizing their retention and peak symmetry on the reversed-phase column. The gradient elution program was as follows: 0–8 min (0% B); 8–15 min (0% → 8% B); 15–21 min (8% → 35% B); 21–22 min (35% → 0% B); and 22–30 min (0% B).

### 2.3. Preparation of PA Composite Gels

In the formulation optimization stage, key parameters including gel matrix type, matrix concentration, glycerol content, HP-β-CD inclusion ratio, and calamine particle size and dosage were systematically screened by single-factor experiments. These were evaluated based on gel formation state, appearance, spreadability, homogeneity, viscosity, and in vitro release behavior to establish the final optimized composition.

Based on these optimized parameters, the final PA composite gel was prepared by a stepwise thermal dissolution and homogenization method. Briefly, 1% (*w*/*w*) Carbomer 940 was uniformly dispersed in 10% (*w*/*w*) glycerol and 10% (*w*/*w*) purified water and allowed to swell in a 65 °C water bath for 2 h. After cooling to room temperature, the dispersion was neutralized with 1% sodium hydroxide solution to obtain a transparent gel matrix (Matrix A). Separately, 8% (*w*/*w*) HP-β-CD and 10% (*w*/*w*) PA extract were dissolved in an appropriate amount of purified water under vigorous stirring to form a homogeneous solution (Solution B). Solution B was then gradually added to Matrix A under continuous stirring.

Subsequently, 4% (*w*/*w*) sodium alginate, 5% (*w*/*w*) calamine powder, and an appropriate amount of preservative were added sequentially. Purified water was then added to achieve 100% of the total formulation weight. The entire mixture was further homogenized using an electric stirrer equipped with a 4 cm diameter impeller for 60 min until a uniform gel system was obtained. The prepared gel was then sterilized and sealed in aluminum tubes for subsequent characterization and biological evaluation.

For comparative evaluations throughout the study, the blank gel was prepared using identical matrix components and homogenization conditions but completely omitting the PA extract and calamine. Similarly, the PA gel control was formulated containing 10% (*w*/*w*) PA extract without the addition of calamine powder.

### 2.4. Characterization of PA Composite Gel

#### 2.4.1. pH Determination of PA Composite Gel

A total of 1 g of PA composite gel was placed in a 50 mL beaker, 10 mL of purified water was added and stirred well, and then the pH value was measured by pH meter.

#### 2.4.2. The Determination of Key Components and HP-β-CD Odour-Masking Effect

HPLC method was employed to ascertain the composition of PA composite gels. Concurrently, GC was used to investigate the olfactory constituents of PA extracts before and after odour masking, investigating the efficacy of HP-β-CD in masking odours.

#### 2.4.3. Study of the Gel Rheological Properties

Steady-state rheological measurements of PA composite gel samples were performed using a cone-plate geometry with a 40 mm diameter, 1.998° cone angle, and 63 μm gap. Shear rate sweeps were conducted. The shear rate determination ranged from 1.5 to 120 s^−1^, which changed in viscosity and sheared rate during determination. During the dynamic rheology experiments, utilize the plate fixture with a diameter of 40 mm. Set the stress to 0.1 Pa and adjust the temperature to 25 °C. Ensure that the frequency varies within the range of 0.1–200 rad/s. Conduct measurements within the linear viscoelastic region, where the elastic modulus is denoted by G′ and the viscous modulus is denoted by G″.

#### 2.4.4. In Vitro Drug Release Study

The in vitro release of uracil from the PA gel and PA composite gel was evaluated using upright Franz diffusion cells with an effective diffusion area of 2.26 cm^2^. A cellulose acetate membrane (0.45 μm pore size) was mounted between the donor and receptor compartments. The receptor compartment was filled with 7.0 mL of phosphate-buffered saline (PBS, pH 7.4) and maintained at 32 ± 0.5 °C with continuous stirring at 300 rpm. An accurately weighed 1.0 g of each gel was evenly spread onto the membrane in the donor compartment. At predetermined time points, 1.0 mL of the release medium was withdrawn from the receptor compartment and immediately replaced with an equal volume of fresh pre-warmed PBS. The uracil concentration in each sample was determined by the validated HPLC method described in Section 2.2, and the cumulative release percentage was calculated.

#### 2.4.5. In Vitro Transdermal Experiments

Skin samples from healthy male Sprague Dawley rats were obtained for in vitro transdermal assays. The upright Franz diffusion cell method was employed with a release area of 2.26 cm^2^, utilizing normal saline as the receiving medium. The volume of the receiving chamber was 7 mL. A total of 1 g of the PA composite gel was evenly distributed on the pretreated intact and exfoliated rat skin, respectively. The speed of the diffusion cell was adjusted to 300 rpm at 32 °C to maintain a dynamic environment within the receiver chamber. At 0.5, 2, 4, 6, 8, 10, 12, and 24 h, 7.0 mL of fluid was extracted from the receiver chamber and replaced with an equal volume of fresh recipient fluid. Subsequently, the drug concentration was determined through HPLC following filtration with a 0.45 μm microporous membrane. The cumulative volume of uracil was calculated and scored in accordance with the following formula:

Q = C_n_/A × V_1_ + ∑C_n−1_ × V_2_ (1)

where C_n_ is the concentration of the sampling point (μg/mL), n is the sampling time points (0.5, 2, 4, 6, 8, 10, 12, and 24 h); V_1_: volume of received liquid in the receiver chamber (mL); V_2_: Volume per sample (mL); A: Effective transmission area (2.26 cm^2^); Q: Cumulative drug penetration per unit area (μg/cm^2^).

### 2.5. Stability Studies of PA Composite Gels

The stability of the PA composite gel was systematically evaluated through centrifugation, high-temperature resistance, and long-term storage tests. In the centrifugation test, 1 g gel samples were subjected to 3000 rpm for 30 min followed by visual inspection for structural integrity. For high-temperature stability test, triplicate samples sealed in storage containers were incubated at 60 °C, with 1 g aliquots collected at 0, 5, and 10 days for quantitative analysis of key components, macroscopic examination, and pH measurement. Similarly, long-term stability was determined by storing sealed samples at 4 °C, where 1 g specimens were periodically retrieved at 0, 15, 30, 60, and 90 days for identical parameter evaluations.

### 2.6. In Vitro Antioxidant and Pro-Migratory Capacities

#### 2.6.1. In Vitro Intracellular ROS Scavenging Assay

Raw264.7 cells were stimulated with 100 μM H_2_O_2_ to induce oxidative stress and subsequently incubated with the hydrogel co-culture medium for 2 h. The intracellular ROS generation levels were then determined using a DCFH-DA fluorescent probe (5 mM) according to the manufacturer’s instructions (Beyotime, Shanghai, China).

#### 2.6.2. In Vitro Scratch Wound Healing Assay

Human umbilical vein endothelial cells (HUVECs) were seeded into 6-well plates at a density of 3 × 10^5^ cells/mL. Following a 24 h incubation to allow for cell adhesion and the formation of a confluent monolayer, a linear scratch was created across the cell layer using a sterile 200 μL pipette tip. The existing medium was discarded, and the scratched monolayers were gently washed with PBS to remove any detached cells and debris. Subsequently, serum-free medium was introduced to sustain the cells and minimize the interference of cell proliferation during the migration process. The migration status of the cells in each group was observed, and images were captured at 0 h and 24 h post-wounding. The wound area was measured using ImageJ software (version 1.53t; National Institutes of Health, Bethesda, MD, USA), and the relative migrated area was calculated using the following formula:

Relative migrated area (%) = (A_0_ − A_t_)/A_0_ × 100% (2)

where A_0_ represents the initial scratch area at 0 h, and A_t_ represents the remaining scratch area at the specific time point.

### 2.7. In Vivo Pharmacodynamic Evaluation

#### 2.7.1. Animals

Male Sprague Dawley (SD) rats (190–210 g) were provided by Liaoning Changsheng Biotechnology Co., Ltd. (Benxi, China). Rats were raised in a standard environment (light cycle 12:12, temperature 25 ± 2 °C, humidity 60 ± 10%). All experiments were approved by the Laboratory Animal Ethical and Welfare Committee of Shenyang Pharmaceutical University (No. SYPU-2ACUC-S2025-0820-208). All animal experiments were performed under the Guide for Care and the Animal Management Rules of the Ministry of Health of the People’s Republic of China.

#### 2.7.2. Establish the Wound Model of Diabetes Rats

Twenty-five adult healthy Sprague Dawley (SD) rats were acclimatized for 7 days in a standardized SPF-grade animal facility. For modeling, streptozotocin (STZ) was dissolved in pre-chilled 0.1 mol/L sodium citrate buffer (pH 4.2) to prepare a 2% (*w*/*v*) fresh solution under light-protected conditions with ice-bath preservation. A single intraperitoneal injection was administered at a dose of 65 mg/kg (injection volume 2 mL/kg), with all injections completed within 15 min post-reconstitution to minimize drug degradation. At 72 h post-injection, fasting blood glucose levels were measured via the glucose oxidase method using tail vein blood samples collected after 6 h fasting. Diabetes mellitus model establishment was considered successful when two consecutive glucose measurements (24 h apart) yielded values ≥ 16.7 mmol/L. This hyperglycemic threshold was adopted because sustained blood glucose above 16.7 mmol/L is known to profoundly impair multiple phases of wound healing, thereby generating a reliable refractory wound model for evaluating therapeutic interventions.

After successful establishment of the diabetic wound model, animals were randomly allocated into different treatment groups using a randomization procedure. Baseline body weight and initial wound area were examined to ensure comparability among groups before treatment. The backs of 25 diabetic rats were depilated. Following an overnight fast, each rat received an intraperitoneal injection of 10% chloral hydrate (300 mg/kg) prior to surgery. A circular wound about 3.0 cm in diameter was designed with the spine of the rat back as the midline [23]. The rats were then randomly assigned to five groups (n = 5): Group A (model control), Group B (Kangfuxin Liquid), Group C (blank gel), Group D (PA gel), and Group E (PA composite gel). Each group received 0.5 g of their assigned treatment twice daily (morning and evening) for 18 consecutive days. Throughout the 18-day treatment period, the fasting blood glucose levels of all diabetic rats remained consistently above 16.7 mmol/L, thereby ensuring the reliability of the chronic diabetic wound model.

#### 2.7.3. Wound Healing Assay

Digital photographs of the wounds were taken on days 0, 6, 12, and 18. Wound areas were quantified using Image-Pro Plus software (version 6.0.0; Media Cybernetics Inc., Rockville, MD, USA). The wound healing rate was calculated as follows:

(3) $$\mathrm{Wound}\;\mathrm{healing}\;\mathrm{rate}\;(\%)\;=\;\frac{\left(A_{0}-A_{t}\right)}{A_{0}}*100\%$$

where “A_0_” indicates the area of the original wound, and “A_t_” is the area of the wound at the measured time point.

#### 2.7.4. Immunohistochemical Testing

Tissue samples were collected at 0, 11, and 18 days post-treatment. Prior to sampling, rats were fasted for 12 h, Wound surfaces and adjacent marginal tissues were excised and fixed with 4% paraformaldehyde. Immunohistochemistry was performed to assess positivity rates of AGEs, RAGE, FGF2, PDGF, and TNF-α in granulation tissue.

#### 2.7.5. Measurement of MDA and SOD

Wound granulation tissues were homogenized in ice-cold PBS and centrifuged at 12,000× *g* for 15 min at 4 °C. The protein concentration of the supernatants was determined using a BCA protein assay kit (Solarbio, Beijing, China). To evaluate the local oxidative stress, the levels of malondialdehyde (MDA) and the activity of superoxide dismutase (SOD) were quantified using commercial assay kits (Solarbio, Beijing, China) according to the manufacturer’s protocols. The results were normalized to the total protein concentration and expressed as nmol/mg protein for MDA and U/mg protein for SOD.

#### 2.7.6. Western Blot Analysis

Tissues were lysed in RIPA buffer supplemented with protease and phosphatase inhibitors. Equal amounts of total protein (quantified via BCA assay) were separated by SDS-PAGE and transferred onto PVDF membranes. After blocking with 5% BSA, the membranes were incubated overnight at 4 °C with primary antibodies against Phospho-NF-κB p65, and NF-κB p65. Following incubation with HRP-conjugated secondary antibodies, the protein bands were visualized using an ECL detection kit. The optical densities were semi-quantified using ImageJ software, and NF-κB activation was expressed as the ratio of p-p65 to total p65.

#### 2.7.7. Histology of Wound Granulating Tissue

On day 18, wound tissue samples were collected for histological analysis. Samples were fixed in 4% paraformaldehyde, paraffin-embedded, sectioned, and stained with hematoxylin and eosin (H&E) to assess inflammatory cell infiltration and vascularization [24].

### 2.8. Safety Studies

A 3 cm × 3 cm area of hair was removed from each side of the dorsal spine of four healthy adult rabbits. One side received a 4 h application of 1 g of PA composite gel; the contralateral side served as a control. Skin reactions were assessed at 1, 24, 48, and 72 h post-treatment.

### 2.9. Statistical Analysis

All results were expressed as mean of three measurements with standard deviation (Mean ± SD). The statistical analysis was performed using GraphPad Prism V 5.0. All parameters were compared using one-way ANOVA. The paired *t*-test was employed to compare the obtained parameters, considering the *p*-value (*p* < 0.05) as statistically significant.

## 3. Results

### 3.1. Preparation of PA Extract

**Figure 2:**
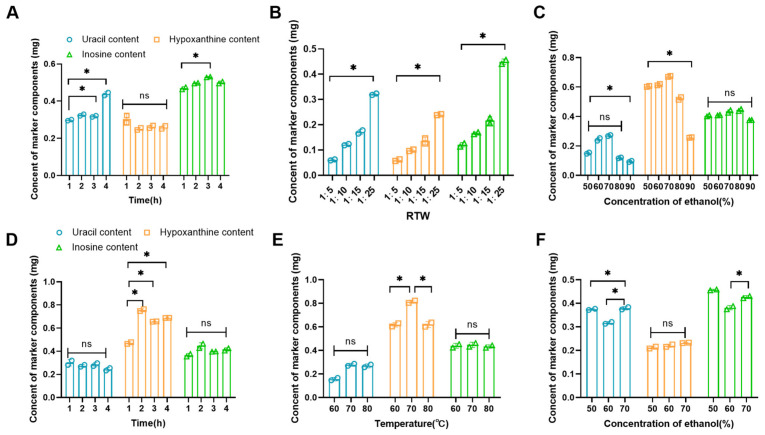
Optimization of the extraction of *Periplaneta americana*. (**A**–**C**) Extraction time, solid–liquid ratio and ethanol concentration on the content of labeled components in ultrasonic-assisted extraction. (**D**–**F**) Extraction time, extraction temperature and ethanol concentration in heating reflux method on content of key components. Data are presented as mean ± SD (n = 3), * *p* < 0.05, and “ns” indicates no significance.

*Periplaneta americana* (PA), an animal-derived traditional Chinese medicine first documented in *Shennong’s Materia Medica*, is pharmacologically significant due to its rich composition of amino acids, peptides, nucleotides, polysaccharides, and other bioactive constituents. Uracil, hypoxanthine, and inosine were selected as quantitative markers for standardization [25,26,27]. This study innovatively integrated traditional processing principles with modern purification technologies to prepare defatted PA extract. Furthermore, this study comparatively evaluated reflux extraction (RE) and ultrasonic-assisted extraction (UAE) methods for PA isolation. Through single-factor experiments with component content as the primary quality indicator, the optimal extraction protocol was established. Under standardized conditions (fixed solid–liquid ratio, extraction duration, and ethanol concentration), UAE demonstrated a 67% reduction in hypoxanthine yield compared to the RE method. Consequently, RE was selected as the preferred PA extraction methodology (Figure 2A–C) [28].

During the RE process, uracil and inosine concentrations remained stable regardless of extraction duration, whereas hypoxanthine content exhibited time-dependent enhancement (*p* < 0.05). The cumulative concentration of these three components peaked at 1.46 mg after 2 h, establishing this duration as optimal (Figure 2D). Temperature screening (60–80 °C) revealed minimal variation in uracil and inosine levels, with maximal hypoxanthine yield (0.804 mg, *p* < 0.05) achieved at 70 °C, thereby defining the optimal extraction temperature (Figure 2E). Given the hydrophilic nature of nucleotides, ethanol concentration markedly influenced extraction efficiency. As shown in Figure 2F, the total component yield demonstrated a parabolic trend, reaching peak values (1.4 mg) at 70% ethanol. The optimized extraction protocol for PA was determined as follows: Powdered raw material was mixed with 70% ethanol (*v*/*v*). The mixture underwent heat reflux extraction at 70 °C for 2 h, with 70% ethanol replenishment to maintain the initial liquid level, ensuring consistent extraction kinetics. The extract was immediately filtered, the filtrate was concentrated until reaching a relative density of 1.15–1.35, yielding a dark brown PA extract paste.

### 3.2. Preparation of PA Composite Gels

Carbomer 940 was identified as the optimal gel matrix through systematic screening of four candidates (carbomer, CMC-Na, gelatin, chitosan), demonstrating superior homogeneity and transparency compared to alternatives forming heterogeneous agglomerates. Glycerol, as a humectant, improves the texture of the gel and helps to lock in the moisture in the skin. The results revealed that with the increase in glycerol content, the viscosity of the gel decreased. When the glycerol content was added to 40%, the gel structure could not be formed. Considering the gel-forming state and retention of the preparation, the ultimate amount of glycerol was determined to be 10% [29,30].

As a traditional Chinese medicine derived from animals, the extract of PA has fishy odour, which is mainly composed of the fatty aldehyde components such as hexanal and isovaleraldehyde. Cyclodextrin can mask the odor through its hydrophobic cavity by forming a 1:1 inclusion complex with the above odorant molecules through hydrophobic interaction. In the previous study, it was found that the results of hypoxanthine and inosine fluctuated greatly during the determination of in vitro transmission, whereas the results of uracil were more stable. Notably, uracil is a recognized biologically active pyrimidine derivative, which can synergistically promote the proliferation of fibroblasts and the maturation of granulation tissue in the wound bed. Therefore, uracil was selected as the representative biomarker for subsequent in vitro release and permeation evaluations.

**Figure 3:**
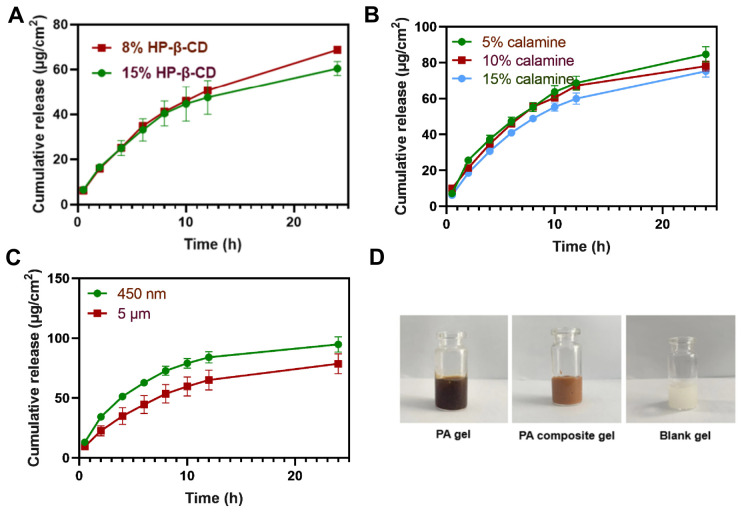
(**A**) Cumulative releases of uracil in gels with different HP-β-CD additions. (**B**) Cumulative releases of uracil in gels with different calamine additions. (**C**) Cumulative releases of uracil in gels with different calamine particle sizes. (**D**) Appearances of PA gel, PA composite gel and blank gel. Data are presented as mean ± SD (n = 3).

In vitro release studies, PA composite gels with different HP-β-CD doses of 8% or 15% demonstrated that the cumulative release of uracil per unit area was 68.67 and 60.43 μg/cm^2^, respectively. It can be seen that increasing HP-β-CD caused a slight decrease. This may be because high concentrations of cyclodextrin may increase the viscosity of the formulation, slowing down the diffusion rate of drug molecules in the medium. Thus, the amount of HP-β-CD added was chosen to be 8% (Figure 3A). In addition, the odour-masking effect of HP-β-CD was investigated by Gas chromatography. When PA extracts were encapsulated by 8% HP-β-CD, the peak areas for hexanal and isovaleraldehyde were reduced to 22.23% and 4.16% of the original peaks, respectively. Thus, it was demonstrated that HP-β-CD could improve the olfactory profile of the extract and serve as a masking agent.

As calamine has the ability to promote eschar formation, its combination with PA extract is expected to have a synergistic effect in the treatment of diabetic wounds. Calamine may alter the viscosity of PA composite gels. This outcome may be attributed to the fact that an excess of calamine incorporated in the powdered form may result in the aggregation of the gel, which subsequently influences the release of the key components. As shown in Figure 3B, the effect of calamine loading (5%, 10%, and 15%) on the cumulative area-specific release of uracil from PA composite gels was investigated. The results demonstrated a decrease in 24 h cumulative release with increasing calamine content. Specifically, the PA gel containing 5% calamine achieved 80.2% drug release within 24 h, whereas the 10% and 15% groups showed reduced releases of 65.1% and 52.4%, respectively. Considering the balance between in vitro release efficiency and drug loading capacity, the optimal calamine content was determined to be 5%. Furthermore, the effect of calamine with particle sizes of 450 nm and 5 μm on the cumulative release of uracil was investigated. Eventually, the 450 nm calamine was ultimately selected based on its superior cumulative uracil release amount per unit area. From this, it can be deduced that the decrease in the particle size of calamine increases the specific surface area of the particles and allows faster drug release rate and higher release volume [31,32] (Figure 3C). The visual presentation of the PA gel, the PA composite gel, and the blank gel is illustrated in Figure 3D. The pH value of the PA composite gel exhibited a range of 6.03 to 6.12.

The PA composite hydrogel was prepared via a stepwise blending method. First, 1% (*w*/*w*) Carbomer 940 was uniformly dispersed in 10% glycerol and 10% purified water, followed by swelling in a 65 °C water bath for 2 h. After cooling to room temperature, the dispersion was neutralized with a 1% NaOH solution to form a transparent gel matrix (Matrix A). Separately, 8% HP-β-CD and 10% PA extract were dissolved in water to form a homogeneous solution (Solution B). Solution B was then gradually incorporated into Matrix A under continuous stirring. Concurrently, 4% sodium alginate, 5% calamine powder, and appropriate preservatives were added. Purified water was added q.s. to 100% of the total formulation weight. The final mixture was homogenized using an electric stirrer (4 cm diameter impeller) for 60 min, sterilized, and sealed in aluminum tubes for subsequent use. Quantitative statistical analysis confirmed that the optimized formulation (8% HP-β-CD and 5% calamine with 450 nm particle size) significantly maximized the cumulative transdermal flux of uracil compared to the other tested sub-optimal ratios, thereby ensuring optimal local drug bioavailability for subsequent tissue repair.

### 3.3. Characterization of PA Composite Gels

#### 3.3.1. Rheological Properties of the PA Composite Gels

**Figure 4:**
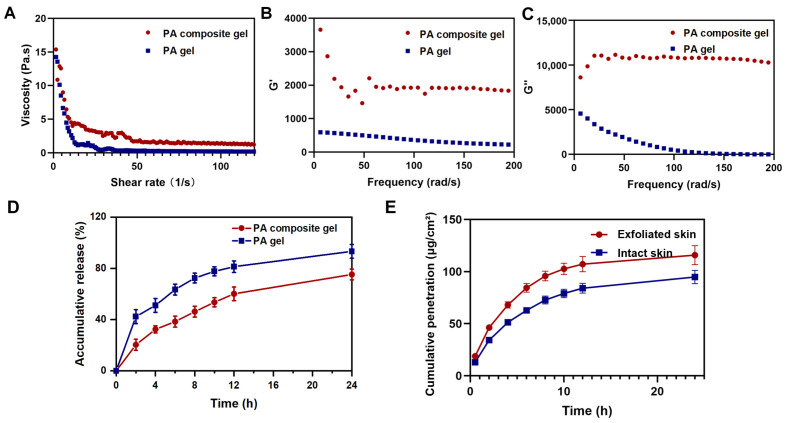
(**A**) Apparent viscosity versus shear rate of PA gel and PA composite gel. (**B**,**C**) G′ and G″ of PA gel and PA composite gel. (**D**) In vitro cumulative release profiles of uracil from PA gel and PA composite gel in PBS (pH 7.4) (n = 3). (**E**) In vitro transdermal permeation profiles of uracil from the PA composite gel through intact and stratum corneum-ablated rat skin (n = 3).

In pseudoplastic fluids, the viscosity of the gel gradually decreases with increasing shear rate, demonstrating characteristic shear-thinning behavior. However, the viscosity of the PA composite gel was consistently higher than that of the PA gel (Figure 4A). This suggests that the incorporation of calamine can increase the viscosity of the gel while maintaining the shear thinning properties of the gel. This gel exhibited satisfactory extensibility on the skin, making it suitable for topical application. The results of the dynamic rheological experiment showed that the elastic modulus (G′) and viscous modulus (G″) of the PA composite gel were consistently higher than those of the PA gel and showed frequency-independent viscoelastic responses. The main reason for this phenomenon was the addition of calamine, which acted as an adhesive on the polymer hydrogel, thereby strengthening the structure and mechanical properties of the gel [33,34] (Figure 4B,C).

#### 3.3.2. In Vitro Drug Release

The cumulative release profiles of uracil from the PA gel and PA composite gel in PBS (pH 7.4) are shown in Figure 4D. Both formulations displayed a biphasic release pattern, with an initial relatively rapid release within the first 4 h, followed by a sustained release phase. At 24 h, the cumulative release of uracil from the PA gel reached 92.2 ± 5.4%, whereas the PA composite gel exhibited a more controlled release of 75.2 ± 4.2%. The lower release rate observed for the PA composite gel is consistent with the incorporation of calamine, which increases matrix density and prolongs the diffusion pathway. This sustained release behavior is favorable for maintaining therapeutic drug concentrations at the wound site over extended periods.

#### 3.3.3. In Vitro Permeability Study of Intact and Exfoliated Skin

Comparative in vitro permeation analysis using intact versus stratum corneum-ablated skin models demonstrated enhanced uracil penetration efficacy in compromised barriers. As quantified in Figure 4E (calculated according to Equation (1)), the composite gel achieved 1.2-fold higher transdermal flux (115.8 vs. 94.8 μg/cm^2^/h) through exfoliated skin, suggesting preferential drug delivery to deeper epidermal layers in chronic wound scenarios. This demonstrated that the PA composite gel may exhibit enhanced permeation in refractory wounds, thereby facilitating drug penetration and significantly increasing effective drug concentration and bioavailability at the affected site [7,35].

### 3.4. Stability Studies of PA Composite Gels

The stability of the PA composite gels was investigated using centrifugal experiment, high-temperature experiments, and long-term storage experiments. Centrifugation experiments showed no signs of phase separation or precipitation, indicating excellent physical homogeneity of the gel matrix.

In the high-temperature stability test, samples were stored at 60 °C and evaluated at days 0, 5, and 10. At each time point, 1 g of gel was sampled to examine appearance, spreadability, and pH, and the contents of uracil, hypoxanthine, and inosine were quantitatively determined by HPLC. The contents of these three marker compounds, which originate from the active PA component in the gel, are reported in mg/g (mass of analyte per gram of gel). After 10 days of high-temperature exposure, the pH of the gel increased by approximately 0.3 units (Table 1). Visual observation revealed a gradual darkening in color and the appearance of a small amount of oil separation, though no distinct layering was observed. Correspondingly, the contents of uracil, hypoxanthine, and inosine decreased by 11.62%, 22.44%, and 21.74%, respectively, relative to the initial values. These changes indicate that the properties of the PA composite gel are susceptible to elevated temperatures.

In the long-term refrigerated storage test at 4 °C, the gel remained visually uniform throughout the 90-day period, with no phase separation or morphological changes (Table 2). The pH was stable during the first 30 days and increased by 0.7 units by day 90. The contents of uracil, hypoxanthine, and inosine were quantified by the same HPLC method and are expressed in mg/g. The retention rates of the three compounds remained between 95% and 105% of the initial values, with a relative standard deviation below 3%, meeting the stability criteria specified in the Chinese Pharmacopoeia. These results confirm that storage at 4 °C effectively preserves the active components of the PA composite gel for at least 90 days.

### 3.5. In Vitro Antioxidant and Pro-Migratory Capacities

**Figure 5:**
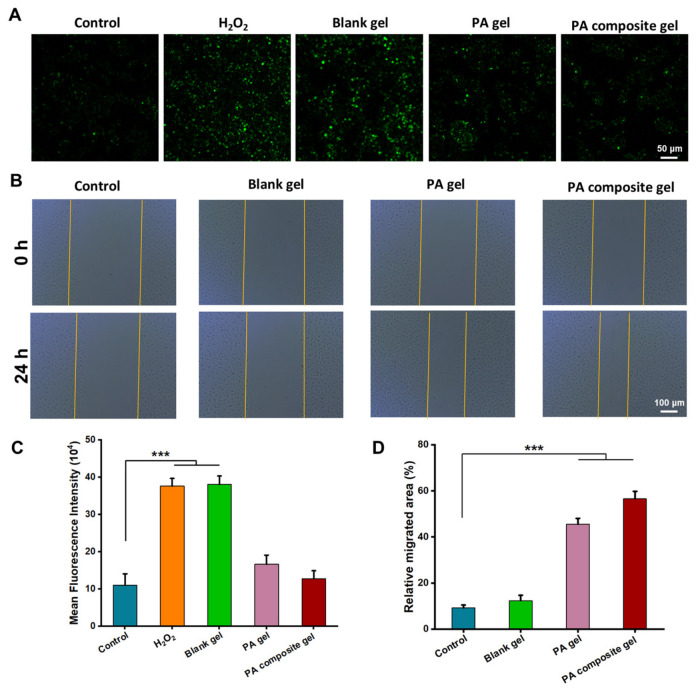
In vitro evaluation of the antioxidant and pro-migratory capacities of the hydrogels. (**A**) Representative fluorescence images of intracellular ROS generation detected by the DCFH-DA probe in H_2_O_2_-stimulated cells following various treatments. Scale bar = 50 μm. (**B**) Representative optical images of the scratch wound healing assay at 0 h and 24 h post-wounding. Scale bar = 100 μm. (**C**) Quantitative analysis of the mean fluorescence intensity of ROS corresponding to panel A (n = 3). (**D**) Quantitative analysis of the relative migrated area (%) corresponding to panel B (n = 3). Data are presented as mean ± SD. *** *p* < 0.001.

Excessive oxidative stress and impaired cellular mobility are major biological barriers that stall diabetic wound healing. To evaluate the microenvironment-modulating ability of the hydrogels, intracellular reactive oxygen species (ROS) levels were first assessed using a DCFH-DA fluorescent probe (Figure 5A,C). Cells exposed to H_2_O_2_ and the blank gel exhibited intense green fluorescence, indicating severe intracellular oxidative stress. In contrast, the PA gel reduced ROS accumulation by 55.6% relative to the H_2_O_2_ group, while the PA composite gel achieved a 65.9% reduction, decreasing ROS to near-basal levels and demonstrating the strongest antioxidant efficacy. This profound ROS clearance suggests that the composite formulation, likely through the synergistic action of PA extracts and zinc ions from calamine, effectively neutralizes the hostile oxidative microenvironment and protects cells from oxidative damage.

Following the mitigation of oxidative stress, the timely migration of fibroblasts is essential for granulation tissue reconstruction. An in vitro scratch assay revealed that the control and blank gel groups exhibited negligible cell motility. In contrast, the PA composite gel significantly accelerated cellular migration into the denuded area, achieving the highest relative migrated area (56.5%, calculated according to Equation (2)) among all groups within 24 h (Figure 5B,D). This superior pro-migratory performance indicates that the PA composite gel not only physically removes the inhibitory oxidative barrier but also actively provides bioactive cues to stimulate cell motility. Collectively, these in vitro findings confirm the dual biomodulatory capacity of the PA composite gel in rescuing cellular functions and accelerating tissue regeneration.

### 3.6. In Vivo Pharmacodynamic Evaluation

#### 3.6.1. Macroscopic Treatment Efficacy in Diabetic Refractory Wounds

**Figure 6:**
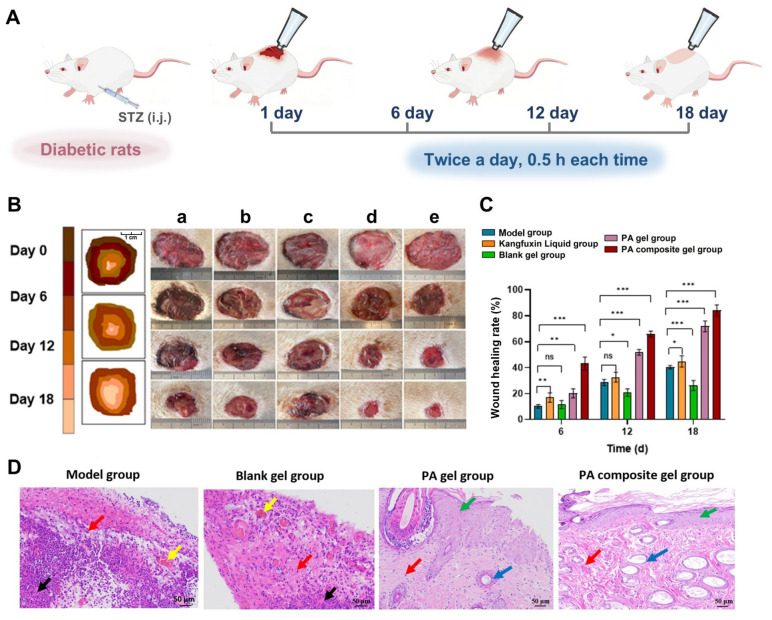
(**A**) Schematic illustration of the establishment of the diabetic rat wound model and the treatment regimen. (**B**) Representative wound photographs from each group on days 0, 6, 12 and 18 for the following groups: (**a**) model group, (**b**) Kangfuxin Liquid group, (**c**) blank gel group, (**d**) PA gel group, and (**e**) PA composite gel group (applicable to all following panels). (**C**) Quantitative analysis of the wound healing rates at days 6, 12, and 18 post-treatment (n = 5). (**D**) Histopathological features of the trauma surface. Black arrows indicate necrotic debris; yellow arrows indicate vascular congestion or dilation; red arrows indicate inflammatory cell infiltration; green arrows indicate epidermal regeneration or re-epithelialization; blue arrows indicate microvascular-like structures. The scale bars are 50 μm. Data are presented as mean ± SD. * *p* < 0.05, ** *p* < 0.01, *** *p* < 0.001, and “ns” indicates no significance.

A streptozotocin (STZ)-induced diabetic rat model with full-thickness cutaneous wounds was established to evaluate the macroscopic therapeutic efficacy of the PA composite gel [36,37]. Each group was treated with 0.5 g of the drug once in the morning and once in the evening, and the treatment was continued for 18 days, ensuring that the drug remained on the wound for more than 30 min (Figure 6A). Complete eschar formation was visually observed across all treatment groups by post-operative day 6 (Figure 6B). Quantitative assessment demonstrated significantly accelerated wound closure rates in the intervention groups compared to the control at day 12: Kangfuxin Liquid (41.51%), PA gel (50.73%), and PA composite gel (57.93%) versus the model group (42.06%) (Figure 6C, calculated according to Equation (3)). This confirms the primary therapeutic contribution of the PA extracts. By day 18, the wound closure rates of the PA gel and PA composite gel groups reached 72.13% and 84.1%, respectively, significantly outperforming the model group. These findings indicate that the integration of calamine promotes rapid wound desiccation and scabbing, exerting a synergistic effect with PA extracts to accelerate the closure of difficult-to-heal wounds [38,39,40].

#### 3.6.2. Histological Remodeling of Wound Granulation Tissue

To further evaluate wound tissue repair, histopathological analysis of the wound beds was performed using H&E staining (Figure 6D). On day 18, the model and blank gel groups showed features associated with delayed healing, including necrotic debris (black arrows), vascular congestion or dilation (yellow arrows), and dense inflammatory cell infiltration (red arrows). In contrast, PA-based formulations, especially the PA composite gel, showed improved histological organization, as indicated by enhanced epidermal regeneration or re-epithelialization (green arrows) and reduced inflammatory cell infiltration compared with the control groups. In addition, the PA composite gel group exhibited increased microvascular-like structures (blue arrows) and more organized fibrous extracellular matrix-like tissue within the granulation tissue. These histological findings suggest that the PA composite gel may alleviate local inflammatory injury and promote granulation tissue formation during diabetic wound repair.

#### 3.6.3. Targeting the AGEs-RAGE Axis to Mitigate Oxidative Stress and Inflammation

Advanced glycation end products (AGEs) and their receptor (RAGE) constitute a pathological axis that chronically entraps diabetic wounds in a state of severe oxidative stress and unresolved inflammation. As the primary upstream trigger, the engagement of AGEs with RAGE activates a signaling cascade that profoundly impairs tissue repair. To elucidate the regulatory mechanism of the PA composite gel on this hostile microenvironment, we comprehensively evaluated the local oxidative burden and inflammatory signaling networks.

**Figure 7:**
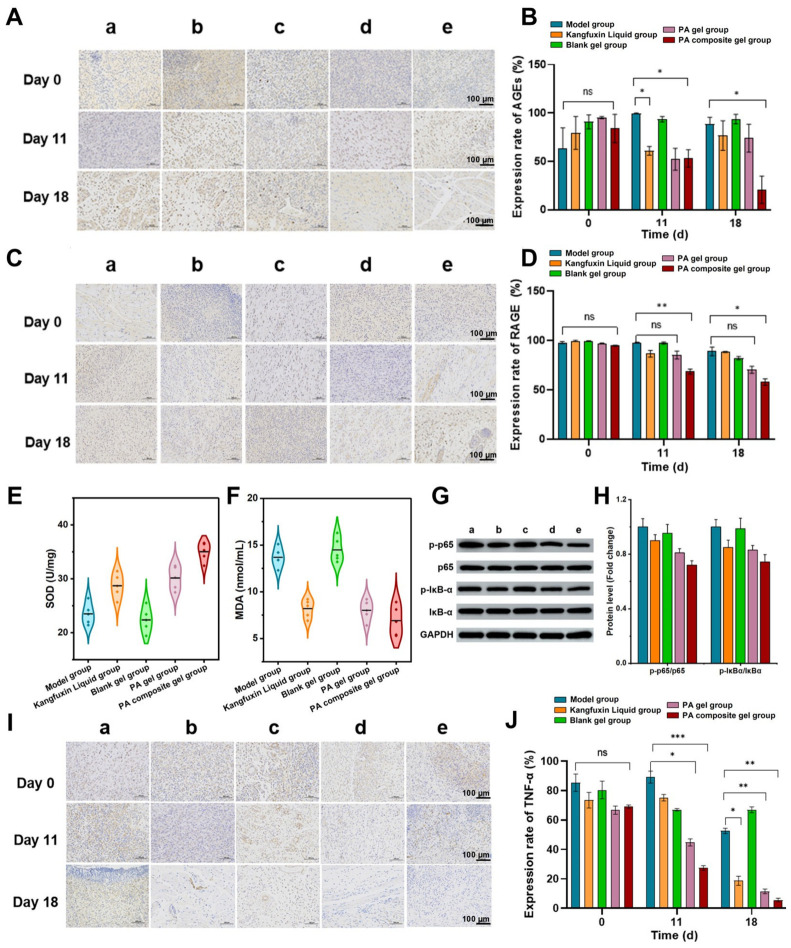
(**A**) Representative images of AGEs expression in the wound granulation tissues of each group: (**a**) Model group, (**b**) Kangfuxin Liquid group, (**c**) Blank gel group, (**d**) PA gel group, and (**e**) PA composite gel group. (**B**) Quantitative analysis of the expression of AGEs (n = 5). (**C**) Expression of RAGE in traumatic granulation tissue in each group. (**D**) Quantitative analysis of the expression of RAGE. Quantitative analysis of (**E**) superoxide dismutase (SOD) activity and (**F**) malondialdehyde (MDA) levels in the wound granulation tissues after 18 days of treatment (n = 5). (**G**) Representative Western blots and (**H**) quantification of p-p65/p65 and p-IκB-α/IκB-α ratios in the wound granulation tissues. (**I**) Expression of TNF-α in traumatic granulation tissue in each group. (**J**) Quantitative analysis of the expression of TNF-α (n = 5). Data are presented as mean ± SD. The scale bars are 100 μm. * *p* < 0.05, ** *p* < 0.01, *** *p* < 0.001, and “ns” indicates no significance.

Longitudinal immunohistochemical profiling demonstrated that the PA composite gel effectively dismantled this upstream trigger. By day 18, the PA composite gel achieved a potent and sustained reduction in AGEs accumulation (74.5% of Model group, Figure 7A,B) and significantly downregulated RAGE expression to 36.8% (Figure 7C,D). Because the AGEs-RAGE interaction is known to drive the overproduction of ROS, we further quantified the local oxidative stress markers. As shown in Figure 7E,F, treatment with the PA composite gel robustly restored superoxide dismutase (SOD) activity, increasing it by 49.1% relative to the model group. Concurrently, it significantly suppressed malondialdehyde (MDA) levels, achieving a 46.3% reduction. This profound antioxidant capacity is likely driven by an observed functional synergy: the organic bioactive molecules in the PA extract directly scavenge free radicals, while the sustained release of Zn^2+^ from calamine likely acts as an essential cofactor to restore the catalytic activity of endogenous Cu/Zn-SOD. Although the explicit Zn^2+^ release kinetics and direct molecular SOD-mimicry require further quantitative validation, the combined formulation significantly outperformed the PA gel alone in mitigating the local oxidative burden.

Crucially, the successful blockade of the AGEs-RAGE/ROS cascade fundamentally paralyzed downstream inflammatory signaling [41,42]. In diabetic wounds, excessive ROS and RAGE activation typically trigger the phosphorylation and nuclear translocation of the transcription factor NF-κB, leading to a cytokine storm. However, Western blot analysis of the wound granulation tissue revealed that the PA composite gel dramatically inhibited the activation of the NF-κB pathway, evidenced by a significantly decreased ratio of phosphorylated p65 (p-p65) to total p65 compared to the model group (Figure 7G,H). This inhibition of NF-κB activation was accompanied by a precipitous drop in the downstream expression of tumor necrosis factor-alpha (TNF-α), which decreased to 5.66% by day 18 (Figure 7I,J). Taken together, these findings rigorously demonstrate that the PA composite gel effectively breaks the vicious cycle of diabetic wound stalling by leveraging the synergistic effects of PA bioactives and calamine-derived Zn^2+^ to physically and biochemically dismantle the AGEs-RAGE/NF-κB axis and neutralize the oxidative microenvironment [43,44].

#### 3.6.4. Upregulation of PDGF and FGF2 to Promote Proliferation and Angiogenesis

Following the successful transition of the stalled diabetic wounds from a state of chronic inflammation to a pro-regenerative microenvironment, the timely activation of tissue reconstruction cascades is imperative for complete healing [45]. Platelet-derived growth factor (PDGF) and basic fibroblast growth factor (FGF2) are master regulators orchestrating this rebuilding stage, driving critical events such as fibroblast proliferation, extracellular matrix (ECM) deposition, and angiogenesis. Therefore, we evaluated the local bioavailability of these vital growth factors within the wound bed via immunohistochemical analysis [46,47].

**Figure 8:**
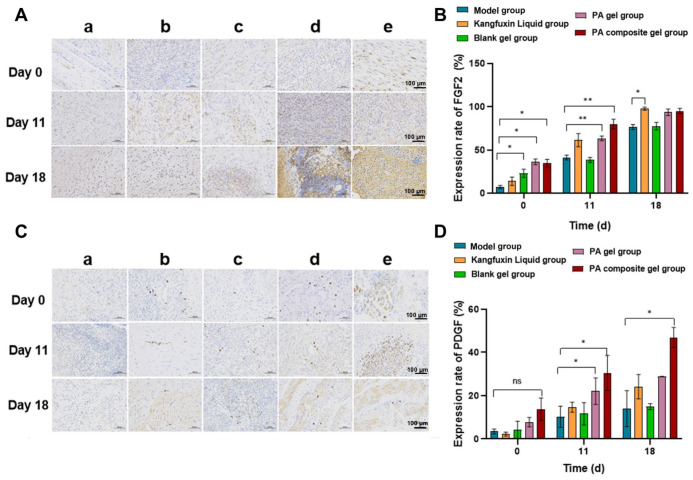
(**A**) Comparison of PDGF in traumatic granulation tissue between groups: (**a**) Model group, (**b**) Kangfuxin Liquid group, (**c**) Blank gel group, (**d**) PA gel group, and (**e**) PA composite gel group. (**B**) Quantitative analysis of the expression of PDGF (n = 5). (**C**) Comparison of FGF2 in traumatic granulation tissue between groups. (**D**) Quantitative analysis of the expression of FGF2 (n = 5). Data are presented as mean ± SD. The scale bars are 100 μm. * *p* < 0.05, ** *p* < 0.01, and “ns” indicates no significance.

Our findings corroborated that the PA composite gel potently amplified the endogenous expression of both regenerative factors. Throughout the treatment period, a progressive upward trend in PDGF and FGF2 immunopositivity was observed in the drug-treated groups compared to the model control. Quantitative analysis revealed that by day 18, FGF2 expression in the PA composite gel group was 18.9% higher than that in the model group, indicating a potent promotional effect (Figure 8A,B). Similarly, PDGF expression was markedly upregulated. The relative PDGF expression in the PA composite gel group was significantly increased by 2.96-fold relative to the model group, which far surpassed the increases observed in the Kangfuxin Liquid group (1.16-fold) and the PA gel alone group (1.34-fold) (Figure 8C,D).

This profound and sustained up-regulation of PDGF and FGF2 provides a compelling molecular rationale for the mature tissue regeneration observed in our previous histological assessments [48]. By harnessing the potent regenerative capacity of PA extracts—which are rich in naturally occurring growth-stimulating peptides—and augmenting it with the influence of calamine-derived Zn^2+^, the PA composite gel effectively revitalizes the proliferative and revascularization potential of the diabetic wound [43,49,50]. This synergistic enhancement directly drives the neo-angiogenesis and organized collagen deposition required to overcome the intrinsic biological barriers of refractory diabetic ulcers, ultimately accelerating robust wound closure.

### 3.7. Safety Studies

**Figure 9:**
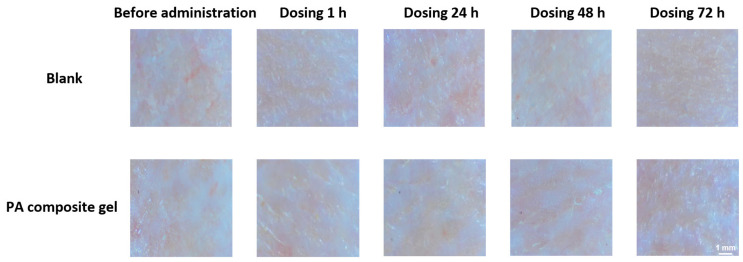
Dorsal skin condition within 72 h after administration (Scale bar = 1 mm).

The PA composite gel showed no erythema or edema during 72 h skin irritation tests compared to blank gel, confirming its safety for damaged wound application (Figure 9).

## 4. Discussion

The clinical management of diabetic foot ulcers (DFUs) is severely compromised by a hostile microenvironment characterized by pathological exudate, persistent oxidative stress, and chronic inflammation. While PA extract possesses well-documented regenerative properties, its conventional liquid clinical formulations (e.g., Kangfuxin Liquid) face inherent limitations in highly exudative diabetic wounds, including rapid clearance, tissue maceration, and poor patient compliance due to volatile odors. In this study, we successfully engineered a synergistic PA-calamine composite hydrogel that transcends passive wound coverage, acting as a dynamic delivery system that physically manages exudate while biochemically reprogramming the stalled wound microenvironment.

### 4.1. Formulation Rationale and Delivery System Advantages

A critical bottleneck in the topical application of PA extracts is the unpleasant olfactory profile driven by fatty aldehydes such as hexanal and isovaleraldehyde. Our formulation utilized HP-β-CD to form hydrophobic inclusion complexes, which successfully reduced the peak areas of these volatile compounds to 22.23% and 4.16%, respectively, significantly improving the formulation’s clinical acceptability. Importantly, in vitro release profiles confirmed that optimizing the HP-β-CD concentration to 8% maintained sustained release of the active marker, uracil, without excessively hindering diffusion.

The incorporation of calamine into the Carbomer 940 matrix provided dual pharmaceutical and biological benefits. Rheologically, the addition of calamine acted as a reinforcing agent, increasing both the elastic (G′) and viscous (G″) moduli. Despite this increased mechanical strength, the composite gel retained critical shear-thinning pseudoplastic behavior, ensuring optimal spreadability upon topical application. Furthermore, optimizing the calamine particle size to 450 nm maximized the specific surface area, facilitating a faster and more comprehensive drug release profile compared to larger microparticles. The targeted delivery capacity of this system was validated by in vitro permeation studies, which demonstrated a 1.2-fold higher transdermal flux through exfoliated skin. This suggests that the hydrogel preferentially delivers active therapeutics into the deeper epidermal layers of compromised chronic wounds, enhancing local bioavailability. Crucially, these optimized physicochemical properties—specifically the shear-thinning applicability, sustained exudate absorption by calamine, and the precisely controlled transdermal flux of active biomarkers—serve as the foundational prerequisite for its in vivo biological performance. By physically shielding the wound bed while dynamically supplying PA bioactives and Zn^2+^, the composite hydrogel establishes an optimal material-tissue interface. Thus, the rational formulation design directly translates material advantages into enhanced tissue repair.

### 4.2. Neutralizing the Hostile Microenvironment: Oxidative Stress and Inflammation

Diabetic wounds are pathologically locked in an inflammatory phase driven by the overproduction of ROS and the accumulation of advanced glycation end products (AGEs). Our in vitro evaluations revealed that the PA composite gel aggressively scavenged intracellular ROS to near-basal levels, outperforming the PA gel monotherapy. This highlights a potent functional antioxidant defense. Rather than isolated intrinsic SOD-like activity, this is likely driven by a functional synergy: PA bioactives directly scavenge free radicals, while calamine-derived Zn^2+^ act as crucial cofactors to replenish endogenous Cu/Zn-SOD activity. By removing this inhibitory oxidative barrier, the composite gel significantly rescued cellular functions, accelerating fibroblast migration to achieve the highest wound closure rate in the scratch assay.

This in vitro microenvironment modulation seamlessly translated to profound in vivo efficacy in the STZ-induced diabetic rat model. The PA composite gel achieved a superior wound closure rate of 84.1% by day 18, visually characterized by rapid desiccation, scabbing, and mature histological remodeling with reduced neutrophil infiltration. Mechanistically, we demonstrated that the formulation achieved this by physically and biochemically dismantling the pathological AGEs-RAGE signaling axis. The composite gel effectively suppressed AGEs accumulation and RAGE expression. Consequently, this upstream blockade mitigated lipid peroxidation (reduced MDA) and restored endogenous antioxidant defenses (elevated SOD).

Consistent with the above findings, the PA composite gel reduced p65 phosphorylation and TNF-α expression, supporting its role in attenuating NF-κB-associated inflammatory responses. These effects may help relieve the chronic inflammatory state that impairs diabetic wound healing.

### 4.3. Promotion of Tissue Reconstruction and Angiogenesis-Associated Repair

Wound healing demands a transition from an inflammatory state to a proliferative phase, orchestrated by key mitogenic signals. The resolution of the toxic microenvironment achieved by our composite gel paved the way for robust tissue reconstruction. Immunohistochemical analysis confirmed a profound, synergistic upregulation of platelet-derived growth factor (PDGF) and basic fibroblast growth factor (FGF2). Specifically, PDGF expression was elevated by 2.96-fold compared to the unhealed model group.

These master regulators are essential for driving fibroblast proliferation, extracellular matrix deposition, and angiogenesis. The sustained bioavailability of these growth factors—stimulated by the PA extract peptides and amplified by the biomodulatory influence of Zn^2+^—strongly supports the pro-regenerative morphological trends, including enhanced microvascular formation and fibrous network deposition, observed in our histological assessments. Accompanied by excellent skin biocompatibility, these findings rigorously validate that the rationally designed PA-calamine composite hydrogel actively coordinates anti-inflammatory regulation with regenerative amplification.

### 4.4. Limitations and Translational Perspectives

Despite the promising therapeutic efficacy demonstrated by the PA composite hydrogel, several limitations of this preclinical study must be acknowledged. First, while the current sample size (n = 5) is sufficient for initial proof-of-concept, future studies with expanded sample sizes are necessary to further validate these findings. Second, while the STZ-induced rat dorsal wound model is widely utilized for standardized evaluations, it inherently lacks the biomechanical stress, chronic friction, and severe ischemia characteristic of human plantar diabetic foot ulcers. Furthermore, although the composite gel exhibited excellent macroscopic infection control, a dedicated bacteria-infected diabetic wound model (e.g., MRSA-infected) is required in future studies to fully substantiate its intrinsic antimicrobial mechanisms. Future studies should incorporate specific histological evaluations, such as Masson’s trichrome and CD31 staining, to definitively confirm collagen organization and neoangiogenesis. Molecularly, further comprehensive transcriptomic analyses via qPCR and deep intracellular kinase profiling are warranted to map the complete downstream cellular transduction networks.

From a translational perspective, Kangfuxin Liquid was utilized as the quantitative clinical standard in this study due to its established status as a first-line therapy for diabetic skin ulcers in China. However, future clinical trials should incorporate quantitative comparisons with Western standard-of-care dressings. Scalability and global commercialization present additional challenges. While calamine is a well-established, easily accessible GMP-grade pharmaceutical excipient, the sourcing of *Periplaneta americana* requires strict adherence to standardized insect farming practices (such as the Jiangsu breeding base utilized here) to minimize batch-to-batch biological variability. Finally, introducing an insect-derived, multi-component traditional medicine into Western regulatory frameworks (e.g., FDA or EMA) poses significant hurdles. Overcoming these barriers will demand rigorous chemical standardization, clear pharmacokinetic profiling of the active markers, and comprehensive toxicological data before this highly synergistic therapy can be globally integrated into DFU clinical management.

## 5. Conclusions

In summary, we first optimized the extraction conditions for PA bioactive components and subsequently developed a composite hydrogel incorporating both the optimized extract and calamine. The PA composite hydrogel demonstrated enhanced skin permeability and shear-thinning rheological behavior, enabling effective delivery of therapeutic agents to diabetic wounds. By synergistically combining the tissue-regenerative capacity of *Periplaneta americana* extract with calamine’s dual functions of wound exudate absorption and inflammatory mediator modulation, the hydrogel exhibited remarkable diabetic wound repair properties. Furthermore, the coordinated action of these components established a dynamic equilibrium between tissue regeneration and anti-inflammatory regulation through simultaneous promotion of angiogenic factors and suppression of inflammatory pathways, ultimately demonstrating superior wound healing efficacy. This integrated therapeutic strategy establishes a novel paradigm for diabetic wound management via a rationally designed material-biological coordination mechanism.

## Figures and Tables

**Table 1:** High temperature stability test results of PA composite gel. Data are presented as mean ± SD (n = 3). * *p* < 0.05, and ** *p* < 0.01 compared with the initial values at day 0.

Indicators	0 d	5 d	10 d
Appearance	Normal color	Color deepening	Color does not differ much from 5 days
	Uniformity	Thinner texture	Rare texture and oily precipitation
	No stratification	No stratification	No significant stratification
pH	6.05 ± 0.09	6.26 ± 0.13	6.36 ± 0.09 *
Uracil (mg/g)	0.3962 ± 0.0078	0.3827 ± 0.0071	0.3550 ± 0.0020 **
Hypoxanthine (mg/g)	0.6246 ± 0.0250	0.4844 ± 0.0038 **	0.4678 ± 0.0008 **
Inosine (mg/g)	0.4565 ± 0.0206	0.3913 ± 0.0035 **	0.3585 ± 0.0432 *

**Table 2:** Long-term stability test results of PA composite gel. Data are presented as mean ± SD (n = 3). * *p* < 0.05 compared with the initial values at day 0.

Indicators	0 d	15 d	30 d	60 d	90 d
Appearance	Moderate color	Normal color	Normal color	Normal color	Normal color
pH	6.03 ± 0.03	6.08 ± 0.05	6.09 ± 0.09	6.13 ± 0.08	6.20 ± 0.07
Uracil (mg/g)	0.3893 ± 0.0131	0.3878 ± 0.0113	0.3869 ± 0.0106	0.3834 ± 0.0091	0.3797 ± 0.0095
Hypoxanthine (mg/g)	0.6790 ± 0.0012	0.6709 ± 0.0030	0.6695 ± 0.0054	0.6673 ± 0.0030	0.6606 ± 0.0026 *
Inosine (mg/g)	0.4359 ± 0.0057	0.4343 ± 0.0251	0.4335 ± 0.0019	0.4328 ± 0.0021	0.4242 ± 0.0019

## Data Availability

The datasets used and/or analyzed during the current study are available from the corresponding author upon request.
